# The Incidence of Itching in Thoracic Epidural Morphine Applications: Can Laboratory Parameters Be Effective in Predicting Itching?

**DOI:** 10.7759/cureus.22257

**Published:** 2022-02-15

**Authors:** Gulay Ulger, Ramazan Baldemir, Musa Zengin, Hilal Sazak, Ali Alagoz

**Affiliations:** 1 Anesthesiology and Reanimation, University of Health Sciences, Ankara Atatürk Chest Diseases and Thoracic Surgery Training and Research Hospital, Ankara, TUR

**Keywords:** morphine, laboratory parameters, thoracic epidural morphine, itching, incidence

## Abstract

Background

Epidural morphine, a powerful analgesic, also causes significant itching in patients. This study aimed to determine the incidence of thoracic epidural morphine-induced pruritus (EMIP) after thoracotomy and to investigate preoperative laboratory parameters for predicting itching in patients who received thoracic epidural morphine (TEM).

Methods

The patients were divided into two groups. The itching (+) group consists of patients who developed itching (n=31). The no-itching (-) / control group (n=31) was selected among patients who did not develop itching after TEM. Preoperative hemogram values, neutrophil/lymphocyte rate (NLR), platelet/lymphocyte rate (PLR), lymphocyte/monocytes rate (LMR), preoperative and postoperative alanine aminotransferase, aspartate aminotransferase, alkaline phosphatase (ALP), gamma-glutamyl transferase values, and whether there was itching in the first 48 hours after surgery were determined.

Results

The incidence of thoracic EMIP after thoracotomy was 7.9%. While preoperative and postoperative ALP was found to be lower in patients with itching compared to those without itching. The cut-off value for preoperative/postoperative ALP was 84.5/53. Decreased white blood cell (WBC) could predict pruritus with a borderline statistical significance.

Conclusions

The incidence of EMIP after thoracotomy was lower compared to other literature data. Infusion of morphine only into the epidural area may cause a low incidence of EMIP. Laboratory parameters ALP and WBC can predict EMIP, but other hemogram parameters, NLR, LMR, and PLR cannot predict EMIP.

## Introduction

Thoracic epidural analgesia (TEA) is a frequently applied method for postoperative pain control, especially in patients undergoing thoracotomy in thoracic surgery operations. Epidural morphine is frequently used for analgesic purposes in these patients. However, morphine, which is a powerful pain reliever and opioid receptor agonist, causes significant itching in patients [1]. The usage of morphine causes histamine release and increases serum histamine [2]. However, pruritus cannot be solely attributed to histamine release.

Itching is an uncomfortable condition for patients. Due to itching, epidural morphine usage and analgesic administration may be interrupted [3, 4]. Despite the frequent use of epidural morphine for postoperative analgesia in patients who underwent thoracotomy, there is scanty data on the incidence of thoracic epidural morphine-induced pruritus (EMIP) after thoracotomy in the literature. Therefore, determining and predicting the incidence of EMIP in TEA applications can positively affect early treatment planning, patient comfort, and analgesic management.

Itching is a symptom caused by the effect of inflammatory mediators. Mast cells, eosinophils, and T cells can also cause itching. Additionally, neutrophils can also produce molecules that can cause itching [5]. Neutrophil/lymphocyte ratio (NLR), platelet/lymphocyte ratio (PLR), and lymphocyte/monocytes ratio (LMR) are considered as indicators of systemic inflammation in patients [6, 7]. Therefore, there can be a correlation between hemogram parameters (such as NLR, LMR, PLR and neutrophil, lymphocyte, eosinophil, etc.) and itching complaints of patients who were administered epidural morphine. This study aims to determine the incidence of pruritus in patients who underwent thoracotomy and received thoracic epidural morphine as well as to investigate the role of preoperative NLR, PLR, LMR, and other laboratory parameters in predicting pruritus in patients treated with thoracic epidural morphine.

## Materials and methods

This study was conducted as a retrospective study in a tertiary thoracic surgery center. After receiving ethics committee approval (Date: 25.05.2021, Number: 2012-KAEK-15/2313), operating room medical records and postoperative pain forms of patients who underwent elective thoracotomy and thoracic epidural analgesia (TEA) between December 2016 and March 2021 were retrospectively reviewed. The following patients were included in the study: those between the ages of 18-80, in the American Society of Anesthesiologists (ASA) physical status I-II-III risk group, and with a body mass index (BMI) between 18.5-35 kg/m^2^.

The patients with the following criteria were excluded: Those under the age of 18 and over the age of 80 years, those who had video-assisted thoracoscopic surgery, whose ASA physical score is IV and above, had a BMI below 18.5 and above 35 kg/m^2^; those who were not inserted thoracic epidural catheters (TEC); those who did not receive standard anesthesia protocol; those who were operated under emergency conditions; with missing data, with severe systemic inflammatory disease, with severe systemic inflammatory disease; usage of constant anti-inflammatory drugs or steroids; receiving intraoperative blood product transfusion; have a chronic itchy disease; who use antihistamine/anti-allergic drugs continuously; and a previous history of allergy.

Patients' age, height, body weight, BMI, gender, ASA physical score, preoperative hemogram values, NLR, PLR, LMR, preoperative and postoperative alanine aminotransferase (ALT), aspartate aminotransferase (AST), alkaline phosphatase (ALP), gamma-glutamyl transferase (GGT) values, performed operation, postoperative visual analog scale (VAS) score, whether there was a complaint of itching in the first 48 hours after surgery, whether the patient was given prophylactic or antihistamine/antiallergic after itching in the perioperative period were determined. NLR was calculated by dividing the absolute neutrophil count by the absolute lymphocyte count. PLR was calculated by dividing the absolute platelet number by the absolute lymphocyte number. LMR was calculated by dividing the absolute lymphocyte number by the absolute monocytes number. VAS evaluation was zero as no pain, 10 as maximum pain. VAS values were measured at the postoperative 1st, 6th, 12th, 24th, and 48th hours. The averages of these values were calculated and the total pain assessment was determined as good: VAS < 4, medium: 4 ≤ VAS < 7, bad: VAS ≥ 7.

After the data were collected, the patients were divided into two groups those with and without itching. Group-I consists of patients who developed itching (n=31). Group-II consists of patients who did not develop itching (n=362). The control group (n=31) was selected from the patients in Group-II (without itching) by a simple random sampling method. The number of men and women was equal to in Group-I.

Anesthesia induction, maintenance, and perioperative analgesia protocol routinely applied in our clinic are as stated in the continuation of the paragraph. For general anesthesia induction, propofol (2 mg/kg), vecuronium (0.1 mg/kg), and fentanyl (1 µg/kg) are administered intravenously. Anesthesia is maintained by administering 2% sevoflurane in the O2-air mixture and remifentanil infusion (0.01-0.20 µg/kg/min). Additionally, intraoperative analgesia is provided by epidural infusion. For epidural analgesia, bupivacaine 0.125% (1.25 mg/ml) and 0.04 mg/ml morphine are infused into the epidural space with an elastomeric pump at a rate of 4 ml/hour for 48 hours postoperatively, starting in the intraoperative period. In the postoperative period, dexketoprofen 50 mg in each every 12 hours, and paracetamol 1 g in every 6 hours are administered intravenously for analgesic purposes.

Statistical analysis

Data were analyzed by the IBM Statistical Package for the Social Sciences for Windows (SPSS), version 22.0 (IBM Corp., Armonk, NY). Whether the distribution of continuous variables was normal or not was determined by Kolmogorov-Smirnov testing. Levene’s test was used for the evaluation of homogeneity of variances. Unless specified otherwise, continuous data were described as mean ± standard deviation (SD) for normal distributions, and median (interquartile range) for skewed distributions. Categorical data were described as a number of cases (%).

Statistical differences in normally distributed variables between two independent groups were compared by Student’s t-test. Mann-Whitney U tests were applied for comparisons of not normally distributed data. Categorical variables were compared using Pearson’s chi-square test or Fisher’s exact test.

One variable logistic regression was used with risk factors that are thought to be related to pruritus. Risk factors that have a p-value < 0.25 as a result in the one variable logistic regression model were included in the multivariable logistic regression model. Whether every independent variable was significant on the model was analyzed with Wald statistic. Nagelkerke R^2^ was used to evaluate how much of the dependent variable can be explained with the independent variables. Besides, a model adaptation of estimates was evaluated with Hosmer and Lemosow model adaptation test. Receiver operating characteristic (ROC) curve analysis was used to determine the cut-off points. Degrees of the relation between variables were evaluated with Pearson correlation or Spearman correlation analysis. P-value <0.05 was accepted as a significant level on all statistical analyses. P-value > 0.05 and p-value < 0.10 was accepted as a marginally significant level on all statistical analysis.

## Results

Between December 2016 and March 2021, 441 patients who underwent elective thoracotomy and TEA were identified. Of these patients, 16 were excluded because they did not meet the inclusion criteria, 17 patients had their epidural catheter removed before 24 hours, and 15 patients were excluded because of missing data. Therefore, a total of 393 patients were analyzed.

Of the remaining 393 patients, 31 (7.9%) had itching complaints during the first 48 hours of follow-up. Morphine infusion was stopped in 21 (67.7%) of the patients with itching, and 10 (32.3%) of the patients with itching were given an antihistamine (pheniramine maleate); these 10 patients comprised 47.6% of the patients whose morphine infusion was stopped. In 10 (32.3%) of the patients with itching, morphine infusion was continued and these patients did not need an antihistamine. Among patients (n=31) with itching, 14 (45.1%) of them in the first 24 hours, six (19.3%) of them in the first 36 hours, and 11 (35.4%) of them in the first 48 hours had developed itching complaints.

Table 1 displays the comparison of the demographic characteristics of patients with and without pruritus. According to the results, there was no statistically significant difference between the groups in terms of age, gender, BMI, type of surgery, ASA physical status, and total pain status (Table 1).

**Table 1:** Patients’ demographic and clinical characteristics Continuous variables are expressed as either the mean ± standard deviation (SD) or median (interquartile range) and categorical variables are expressed as frequency (percentage). Continuous variables were compared with Student’s t-test *. Categorical variables were compared using Pearson’s chi-square test or Fisher’s exact test β. BMI: Body mass index, SD: Standard deviation, ASA: American Society of Anesthesiologists, IQR: interquartile range

		Itching (+) (n:31)	Itching (-) (n:31)	Total (n:62)	p-value
Age, ± SD		52.16 ± 14.01	52.52 ± 13.75	52.34 ± 13.77	0.920*
Gender, n(%)					
	F	7 (22.6%)	7 (22.6%)	14 (22.6%)	0.999β
	M	24 (77.4%)	24 (77.4%)	48 (77.4%)	
BMI, ± SD		25.27 ± 4.02	25.46 ± 3.77	25.37 ± 3.86	0.851*
Operation, n(%)					
	Segmentectomy / lobectomy	26 (83.9%)	27 (87.1%)	53 (85.5%)	0.429β
	Pneumonectomy	2 (6.5%)	4(12.9%)	6 (9.7%)	
	Decortication	2 (6.5%)	-	2 (3.2%)	
	Hydatid cyst / cystectomy	1 (3.2%)	-	1 (1.6%)	
ASA, n(%)					
	1	1 (3.2%)	-	1 (1.6%)	0.797 β
	2	13 (41.9%)	12 (38.7%)	25 (40.3%)	
	3	17 (54.8%)	19 (61.3%)	36 (58.1%)	
Global Pain, n(%)					
	Good	18 (58.1%)	25 (80.6%)	43 (69.4%)	0.054 β
	Medium	13 (41.9%)	6 (19.4%)	19 (30.6%)	
	Bad	-	-	-	
Using antihistamine (pheniramine maleate) for Itching, n(%)		10 (32.3%)	-	-	-
Morphine infusion stop, n(%)		21 (67.7%)	-	-	-
Itching start time (hour), median (IQR)		36 (24)	-	-	-

There is no statistically significant difference between the groups in terms of hemogram parameters (Table 2).

**Table 2:** NLR, LMR, PLR values, and hemogram parameters of the patients Continuous variables are expressed as either the mean ± Standard deviation (SD) or median (interquartile range). Continuous variables were compared with Student’s t-test * or Mann-Whitney U test Φ. IQR: interquartile range, LMR: lymphocyte/monocyte rate, MCV: mean corpuscular volume, MCH: mean corpuscular hemoglobin, MCHC: mean corpuscular hemoglobin concentration, MPV: mean platelet volume, NLR: neutrophil /lymphocyte rate, PCT: platelet crit, PDW: platelet distribution width, PLR: platelet/lymphocyte rate, RBC: red blood cell, RDW: red cell distribution width, SD: standard deviation, WBC: white blood cell

	Itching (+) (n:31)	Itching (-) (n:31)	Total (n:62)	p-value
NLR, median (IQR)	2.29 (2.22)	2.13 (1.43)	2.22 (1.57)	0.949 Φ
LMR, median (IQR)	3.87 (3.33)	3.82 (3.04)	3.85 (3.04)	0.871 Φ
PLR, median (IQR)	145.97 (121.91)	117.11 (73.24)	128.10 (97.83)	0.688 Φ
WBC, median (IQR)	6.66 (3.42)	7.31 (3.76)	7.31 (3.31)	0.087 Φ
Lymphocyte, ± SD	1..93 ±0.87	2.20 ±0.75	2.07 ±0.82	0.205 *
Monocytes, median (IQR)	0.47 (0.34)	0.55 (0.25)	0.52 (0.27)	0.195 Φ
Neutrophil, median (IQR)	3.91 (2.47)	4.53 (2.96)	4.21 (2.79)	0.110 Φ
Eosinophil, median (IQR)	0.13 (0.19)	0.16 (0.20)	0.16 (0.20)	0.360 Φ
Basophil, median (IQR)	0.04 (0.05)	0.05 (0.03)	0.05 (0.04)	0.303 Φ
RBC, median (IQR)	4.78 (0.86)	4.70 (0.97)	4.73 (0.88)	0.894 Φ
Hemoglobin, median (IQR)	13.60 (3.00)	13.60 (3.00)	13.60 (2.90)	0.882 Φ
Hematocrit, median (IQR)	42.50 (8.90)	41.30 (8.50)	41.70 (8.80)	0.741 Φ
MCV, median (IQR)	89.70 (4.30)	88.10 (5.20)	89.20 (4.40)	0.730 Φ
MCH, median (IQR)	29..10 (2.50)	29.50 (2.20)	29.30 (2.20)	0.434 Φ
MCHC, median (IQR)	32.70 (1.90)	32.90 (1.20)	32.80 (1.50)	0.250 Φ
RDW, median (IQR)	14.80 -3	15.30 (4.20)	14.90 (3.30)	0.176 Φ
Thrombocyte, median (IQR)	235 -108	261 -126	247 -120	0.125 Φ
MPV, ± SD	8.06 ±1.10	8.00 ±1.41	8.03 ±1.26	0.830 *
PCT, ± SD	0.21 ±0.08	0.22 ±0.06	0.22 ±0.07	0.395 *
PDW, ± SD	16.85 ±1.33	16.94 ±0.98	16.89 ±1.16	0.779 *

Preoperative ALP and postoperative ALP are statistically significantly lower in the patients with itching than in those without itching (Table 3).

**Table 3:** Preoperative and postoperative bilirubin, ALP, ALT, AST, and GGT values of the patients Continuous variables are expressed as median (interquartile range). Continuous variables were compared with Mann-Whitney U test Φ. ALP: alkaline phosphatase, ALT: alanine aminotransferase, AST: aspartate aminotransferase, BIL: bilirubin, GGT: gamma-glutamyl transferase, IQR: interquartile range, Preop: preoperative, Postop: postoperative

	Itching (+) (n:31)	Itching (-) (n:31)	Total (n:62)	p-value
Preop, direct BIL	0.16 (0.16)	0.17 (0.09)	0.17 (0.12)	0.442
Preop, indirect BIL	0.31 (0.24)	0.36 (0.31)	0.37 (0.26)	0.833
Postop, direct BIL	0.15 (0.23)	0.26 (0.15)	0.27 (0.16)	0.944
Postop, indirect BIL	0.45 (0.30)	0.51 (0.43)	0.49 (0.37)	0.632
Preop ALP, median (IQR)	72 (21)	87 (37)	82 (29)	0.015 Φ
Preop ALT, median (IQR)	17 (19)	15 (13)	15.5 (15)	0.159 Φ
Preop AST, median (IQR)	21 11)	17 (11)	19 (11)	0.102 Φ
Preop GGT, median (IQR)	24.5 (25)	28 (19)	25 (21)	0.363 Φ
Postop ALP, median (IQR)	57 (23)	66 (26)	62 (25)	0.022 Φ
Postop ALT, median (IQR)	21 (22)	16 (19)	18 (19)	0.272 Φ
Postop AST, median (IQR)	32 (17)	30 (25)	30.5 (22)	0.910 Φ
Postop GGT, median (IQR)	21 (19)	20 (31)	21 (21)	0.402 Φ

Logistic regression analysis was applied to determine the factors affecting itching in the patients included in the study (Table 4).

**Table 4:** Univariate and multivariate regression analysis ALP: alkaline phosphatase, ALT: alanine aminotransferase, ASA: American Society of Anesthesiologists, AST: aspartate aminotransferase, BMI: body mass index, CI: confidence interval, GGT: gamma-glutamyl transferase, LMR: lymphocyte/monocyte rate, LR: likelihood ration, MCV: mean corpuscular volume, MCH: mean corpuscular hemoglobin, MCHC: mean corpuscular hemoglobin concentration, MPV: mean platelet volume, NLR: neutrophil/ lymphocyte rate, OR: odds ratio, PCT: platelet crit, PDW: platelet distribution width, PLR: platelet/lymphocyte rate, Postop: postoperative, Preop: preoperative, RBC: red blood cell, RDW: red cell distribution width, Wald: test statistics, WBC: white blood cell

	Univariate Logistic Regression				Multivariate Logistic Regression			
					(Backward LR 5. Step)			
	Wald	p	OR	95% GA	Wald	p	OR	95% GA
Age	0.01	0.919	0.998	(0.962-1.035)				
Gender	0	0.999	0.999	(0.304-3.289)				
BMI	0.037	0.848	0.987	(0.867-1.124)				
ASA	0.519	0.471	0.705	(0.272-1.826)				
NLR	0.017	0.897	1.012	(0.851-1.202)				
LMR	0.207	0.649	0.967	(0.836-1.118)				
PLR	1.357	0.244	1.004	(0.998-1.010)				
WBC	3.499	0.061	0.839	(0.699-1.008)	3.087	0.079	0.831	(0.676-1.022)
Lymphocyte	1.595	0.207	0.658	(0.344-1.260)				
Monocytes	0.129	0.72	0.678	(0.081-5.665)				
Neutrophil	2.341	0.126	0.853	(0.696-1.046)				
Eosinophil	0.35	0.554	1.459	(0.417-5.101)				
Basophil	0.259	0.611	1.475	(0.330-6.587)				
RBC	0.433	0.511	1.131	(0.783-1.634)				
Hemoglobin	0.494	0.482	1.036	(0.938-1.144)				
Hematocrit	0.659	0.417	1.011	(0.985-1.038)				
MCV	0.767	0.381	0.971	(0.910-1.037)				
MCH	0.949	0.33	0.909	(0.751-1.101)				
MCHC	0.371	0.542	1.028	(0.941-1.123)				
RDW	1.972	0.16	0.846	(0.670-1.068)				
Thrombocyte	1.07	0.301	0.997	(0.991-1.003)				
MPV	0.048	0.827	1.046	(0.701-1.559)				
PCT	0.738	0.39	0.043	(0.00-57.006)				
PDW	0.082	0.774	0.938	(0.608-1.449)				
Preop ALP	3.777	0.052	0.981	(0.963-1.000)	5.583	0.018	1.06	(1.010-1.112)
Preop ALT	1.375	0.241	1.022	(0.985-1.060)				
Preop AST	0.078	0.781	1.006	(0.964-1.050)				
Preop GGT	0.001	0.973	1	(0.988-1.013)				
Postop ALP	4.516	0.034	0.97	(0.943-0.998)	6.787	0.019	0.956	(0.924-0.989)
Postop ALT	0.498	0.48	1.006	(0.989-1.024)				
Postop AST	0.772	0.38	0.994	(0.981-1.007)				
Postop GGT	0	0.992	1	(0.987-1.013)				

As a result of the one variable logistic regression analysis, the factors that were determined to have a statistical effect on itching (p-value<0.25) were identified. These variables were included in the multivariate logistic regression analysis, while the variables that did not have a significant relationship were excluded from the analysis. In the multivariate logistic regression analysis, the backward likelihood ratio (LR) model was applied. According to the results of multivariate logistic regression analysis, an increase in preoperative ALP level (odds ratio=1.060, p=0.0018) and a decrease in postoperative ALP level (odds ratio=0.956, p=0.0019) predict pruritus and were found to be statistically significant. Decreased white blood cell (WBC) count (odds ratio=0.831, p=0.079) predicted pruritus and was found to be statistically borderline significant (0.05 < p < 0.10) (Table 4).

To answer the question of which value should be taken as the cut-off for preoperative and postoperative ALP values, the given sensitivity and specificity values were examined and optimum points were selected with the ROC analysis. When the cut-off value for preoperative ALP was accepted as 84.5, the sensitivity was calculated as 74.2% and the specificity as 61.3%. When the cut-off value for postoperative ALP was accepted as 53, the sensitivity was calculated as 48.4% and the specificity as 83.9% (Table 5).

**Table 5:** ROC analysis for preoperative and postoperative ALP ALP: alkaline phosphatase, AUC: area under the ROC curve, CI: confidence interval, ROC: receiver operating characteristic, SE: standard error, Sens: sensitivity, Spec: specificity

	AUC	SE	p-value	95% CI	CutOff	Sens.	Spec.
Preoperative ALP	0.679	0.07	0.015	(0.543-0.815)	84.5	74.20%	61.30%
Postoperative ALP	0.669	0.069	0.022	(0.533-0.805)	53	48.40%	83.90%

## Discussion

In our study, the incidence of itching due to thoracic epidural morphine application after thoracotomy was 7.9%. Preoperative ALP and postoperative ALP were lower in those with itching than in those without. However, according to the results of multivariate logistic regression analysis, an increase in preoperative ALP level (OR=1.060, p=0.0018) and a decrease in postoperative ALP level (OR=0.956, p=0.0019) predict itching. The cut-off values for preoperative and postoperative ALP were determined as 84.5 and 53. The decrease in white blood cell count (OR=0.831, p=0.079) predicted itching at the borderline statistically significantly. It was determined that NLR, PLR, LMR, and other laboratory parameters were not effective in predicting pruritus associated with thoracic epidural morphine administration.

Although different mechanisms have been suggested, morphine can cause itching as a side effect, and itching seriously impairs patient comfort [1-4, 8]. Morphine is a frequently preferred agent in epidural applications, especially for postoperative analgesia. Therefore, EMIP is of clinical interest. However, EMIP studies were mostly performed on patients who delivered by cesarean section [9-13]. In these studies, the incidence of EMIP ranged from 18.6% to 73%. In our study, the incidence of thoracic EMIP was found to be 7.9%. The incidence of EMIP was lower than the literature data. Due to the interaction of estrogen with opioid receptors, pregnant women are more sensitive to neuraxial opioids. This sensitivity can be the reason for the high incidence of EMIP in pregnant women [14].

In the literature, the incidence of EMIP has been reported at different epidural morphine doses and volumes. In a study, 2 mg morphine was given to two different groups as a single dose in 2 ml and 10 ml volumes. 15.7% of those given low volume (2 ml) experienced itching, while 22.7% of those given high volume (10 ml) experienced itching, and the overall incidence of pruritus was 18.6 [9]. In a different study, a single dose of 1.5 mg and 3 mg was given and the incidence of EMIP was reported as 20% and 50% respectively [12]. In another study performed in pregnant women, epidural morphine was given as bolus and infusion, 0.06 mg/ml morphine was administered to the epidural area as 10 ml bolus and 3 ml/hour infusion. According to the result of this study, the incidence of EMIP was reported as 73% at the 24th hour, 62% at the 36th hour, and 37% at the 48th hour of the 36-hour morphine infusion [13]. Similarly, in our study, itching due to epidural morphine was observed most frequently in the first 24 hours of the infusion (45.1% of patients with itching).

In the literature, morphine was given to the epidural area either as a bolus or as a bolus and infusion. In our study, unlike the literature, morphine was given to the epidural area only as an infusion. In our study, morphine infusion was given as 0.16 mg/hour. Similarly, a study gave an epidural morphine infusion dose of 0.18 mg/hour in addition to 0.6 mg bolus morphine [13]. Therefore, the low incidence of pruritus in our study can also be attributed to the infusion of morphine only. With prospective studies, evaluation of the incidence of pruritus due to single-dose morphine and morphine infusion applications should be continued to provide details.

Predicting EMIP in thoracic surgery is very important in terms of establishing healthy and consistent patient treatment protocols. Postoperative severe pain is a frequently encountered condition in patients undergoing thoracotomy. In our study, morphine infusion was stopped in 67.7% of the patients who developed EMIP. This situation causes the interruption of the analgesic treatment of the patients. Therefore, predicting EMIP in advance and taking necessary precautions can be very important in patients undergoing thoracotomy.

There are studies in the literature to determine various risk factors that affect the incidence of EMIP, such as ethnic group, VAS, allergy history, and morphine dose [9]. However, there is no study in the literature about whether EMIP can be predicted objectively with a laboratory parameter. When the literature data is examined, we see that NLR is used as an indicator of diagnosis, prognosis, and inflammation in many clinical situations [6, 7, 15, 16]. In our study, we aimed to evaluate the relationship of EMIP with ALT, AST, ALP, and GGT, which evaluates hemogram parameters including inflammatory and allergic markers and liver functions. Since liver dysfunctions may be associated with itching, we also investigated liver function tests in our study. In our study, preoperative ALP and postoperative ALP were statistically significantly lower in patients with itching compared to those without itching. According to the results of the multivariate logistic regression analysis, an increase in the preoperative ALP level (OR=1.060, p=0.0018) and a decrease in the postoperative ALP level (OR=0.956, p=0.0019) predicted itching and were found to be statistically significant. Therefore, it can be thought that the change in liver functions may be related to EMIP. However, according to the results of our analysis, there was no difference between the two groups in terms of preoperative and postoperative bilirubin, ALT, AST, and GGT values. This indicates that isolated ALP values and pruritus may be related independently to liver functions. In our study, the cut-off values for preoperative and postoperative ALP were determined as 84.5 and 53. These cut-off values, which were determined as a first, may contribute to the literature for future studies that will investigate the relationship between thoracic EMIP and ALP.

In the regression analysis, a decrease in WBC (OR=0.831, p=0.079) appears to be associated with EMIP. However, this situation should be supported by different studies since it is statistically borderline significant. It was determined that NLR, PLR, LMR, and other laboratory parameters were not effective in predicting pruritus associated with thoracic epidural morphine administration.

There are some limitations to our study. First of all, since this study was conducted retrospectively, data such as smoking and alcohol habits of the patients, where the itching is in the body and its prevalence in the body could not be analyzed properly. In addition, the relatively low number of patients with itching negatively affects the power analysis of the results.

## Conclusions

In conclusion, the incidence of EMIP in patients who underwent thoracotomy was found to be 7.9% in our study. This result was observed to be lower than the incidence of EMIP reported in the literature. We think that only infusion of morphine into the epidural area may cause a low incidence of EMIP. We think that among the laboratory parameters, ALP (preoperative/postoperative ALP cut-off value of 84.5/53) and WBC can predict EMIP, while other parameters and NLR, LMR, and PLR cannot predict EMIP.

## References

[1] Wong LS, Wu T, Lee CH (2017). Inflammatory and noninflammatory itch: implications in pathophysiology-directed treatments. Int J Mol Sci.

[2] Rawal N, Schött U, Dahlström B, Inturrisi CE, Tandon B, Sjöstrand U, Wennhager M (1986). Influence of naloxone infusion on analgesia and respiratory depression following epidural morphine. Anesthesiology.

[3] McDonnell NJ, Keating ML, Muchatuta NA, Pavy TJ, Paech MJ (2009). Analgesia after caesarean delivery. Anaesth Intensive Care.

[4] Chaney MA (1995). Side effects of intrathecal and epidural opioids. Can J Anaesth.

[5] Hashimoto T, Rosen JD, Sanders KM, Yosipovitch G (2018). Possible role of neutrophils in itch. Itch (Phila).

[6] Karaca O, Dogan G (2020). Can neutrophil-to-lymphocyte or platelet-to-lymphocyte ratio be used to predict postoperative nausea and vomiting in breast reduction?. Cureus.

[7] Shimoyama Y, Umegaki O, Agui T, Kadono N, Minami T (2017). Neutrophil to lymphocyte ratio and platelet to lymphocyte ratio are superior to other inflammation-based prognostic scores in predicting the mortality of patients with gastrointestinal perforation. JA Clin Rep.

[8] Nguyen E, Lim G, Ding H, Hachisuka J, Ko MC, Ross SE (2021). Morphine acts on spinal dynorphin neurons to cause itch through disinhibition. Sci Transl Med.

[9] Tan X, Shen L, Wang L, Labaciren Labaciren, Zhang Y, Zhang X, Huang Y (2019). Incidence and risk factors for epidural morphine induced pruritus in parturients receiving cesarean section: A prospective multicenter observational study. Medicine (Baltimore).

[10] Dualé C, Frey C, Bolandard F, Barrière A, Schoeffler P (2003). Epidural versus intrathecal morphine for postoperative analgesia after Caesarean section. Br J Anaesth.

[11] Yeh HM, Chen LK, Lin CJ (2000). Prophylactic intravenous ondansetron reduces the incidence of intrathecal morphine-induced pruritus in patients undergoing cesarean delivery. Anesth Analg.

[12] Singh SI, Rehou S, Marmai KL, Jones AP (2013). The efficacy of 2 doses of epidural morphine for postcesarean delivery analgesia: a randomized noninferiority trial. Anesth Analg.

[13] Chen MK, Chau SW, Shen YC (2014). Dose-dependent attenuation of intravenous nalbuphine on epidural morphine-induced pruritus and analgesia after cesarean delivery. Kaohsiung J Med Sci.

[14] Kumar K, Singh SI (2013). Neuraxial opioid-induced pruritus: An update. J Anaesthesiol Clin Pharmacol.

[15] Zhang Y, Lu JJ, Du YP, Feng CX, Wang LQ, Chen MB (2018). Prognostic value of neutrophil-to-lymphocyte ratio and platelet-to-lymphocyte ratio in gastric cancer. Medicine (Baltimore).

[16] Eryigit U, Altunayoglu Cakmak V, Sahin A, Tatli O, Pasli S, Gazioglu G, Karaca Y (2017). The diagnostic value of the neutrophil-lymphocyte ratio in distinguishing between subarachnoid hemorrhage and migraine. Am J Emerg Med.

